# Clinic Atlanta Medical College

**Published:** 1878-04

**Authors:** S. W. Johnson


### CLINICAL LECTURE IN ATLANTA MEDICAL COLLEGE—SERVICE OF DR. J. G. WESTMORELAND.

REPORTED BY S. W. JOHNSON, M.D.

J. M., (white) æt. 33 years, and farmer by occupation.

*Gentlemen*—This man is a fair representative of a variety of indigestion not very frequently met with, but exceedingly difficult to manage successfully. You have witnessed the critical examination to which he has just been subjected, and have probably come already to some conclusions for yourselves.

The sense of weight and fullness in the stomach, red tongue, etc., indicate an irritable condition of the gastric mucous membrane. The increase of pain and sense of heat after taking food confirms the opinion, and leads to the impression that all his trouble is the result of chronic inflammation in the stomach, extending, perhaps to tissues beneath the mucous coat. The uneasiness and fullness exhibited in the right hypochondrium do not tend to change my opinion in this particular, but rather to confirm it. It is a fact constantly verified by experience that organic lesion of the stomach almost invariably leads to hepatic disturbance, and often of such character as to leave doubt as to the organ primarily affected. The same sympathetic impression is made upon the stomach by organic disease of the liver, so that the student of pathology finds great difficulty sometimes in determining the origin of trouble existing in the digestive apparatus. Not only so, but when the location is ascertained, much difficulty is sometimes experienced in arriving at correct conclusions as to the nature of the lesion.

Evidences of a want of proper preparation of food are found in various pathological states of the stomach, and as the result of nervous, hepatic and pancreatic lesions, either organic or functional, as well as primary disease of the stomach itself. Distressing indigestion sometimes exists as the result of enervation of the stomach on account of reflex influence from urinary and uterine disease, transmitted to that portion of the spinal cord supplying the digestive organs.

Sedentary habits also lead to an inactive condition of the muscular and follicular structure of the whole alimentary canal, upon which a form of indigestion with constipation depends. While we have no well established opinion of the use of bile in the process of digestion, certain it is that this fluid is absolutely necessary to the preparation of the food for assimilation, and that inactivity of the liver produces indigestion independently of sympathy existing between the liver and the stomach, before alluded to.

The case before us to-day, gentlemen, is evidently one of organic lesion, as before stated. The deep-red color of the tongue, and the sense of heat and pain after taking food, make this different from the other forms of indigestion, just mentioned, and control me in the opinion I entertain of its inflammatory character. The pathological state in this case is exactly opposite of that caused by sedentary habits, and yet failure of digestion is the result of both. Instead of a want of proper kneading, the food is forced through the pylorus unchimified in the case before us. The inflammation affecting also the glandular bodies which secrete gastric fluid, lessens or vitiates this important solvent of the food, so that the preparation is imperfect on this account.

I desire to direct your attention to the mental disturbance in this case. It is not only an attendant of organic disease of the stomach, but of the liver also, and even in those cases of functional derangement from sedentary habits or nervous origin. This man has forebodings of evil to come upon him, loss of memory, and various forms of imaginary distress. It is called melancholia, or hypochondria. The ancients thought the former an appropriate name, on account of the constancy of dark discharges from the bowels in such cases, which they supposed depended on *melas chole*, black bile. Hence the name, melancholia, or melancholy, indicating a state of mental depression, often attendant upon hepatic and gastric disturbance.

This patient being of sanguine temperament, his florid complexion and general healthy appearance would very likely give the impression that his trouble is, for the most part, imaginary, and that the organ of the mind is the seat of primary disturbance. The mental abberation differs, however, very materially from mania. There is in his case no settled delusion upon which he acts constantly. His dread of evil, though uncontrollable, he knows is imaginary, and in this it differs from insanity.

The treatment of indigestion is as varied, and more so, than the causes which produce it. Cases apparently identical are not always subject to the same course of management. A leading feature in the treatment of all cases, is dieting, and as in this, rare beefsteak is readily digested by some subjects, while others cannot tolerate meats of any kind. Notwithstanding, then, the importance of dieting, there is scarcely a rule on the subject without exceptions. Experience with the particular patient is the only safe criterion. It may be set down as a general rule, that bland, unirritating, nourishing articles of food are more appropriate in cases like this, with tender and irritable gastric surface. Liquids containing the necessary ingredients would seem to be especially appropriate, as they give less mechanical irritation, and require less effort of the stomach for their preparation than solids. Sweet milk being of this kind, would seem to be a suitable article for such cases. Most patients entertain the erroneous idea that perfect nourishment cannot be furnished without solid food, while it is true that very little is afforded by many solid articles, particularly when, from their nature and the condition of the stomach, they are not digested. In fact, under such circumstances, they prove a source of embarrassment, and the patient had better take no food at all for a time.

Relief from the distressing symptoms in this case can be afforded in a few weeks by proper dieting and counter-irritation over the epigastrum. It must be remembered, however, that, like chronic inflammation in other parts, there will be constant tendency to its return after having entirely subsided. Care in guarding against irritating causes and watchfulness in order to detect and arrest the return of inflammation, which is certain to occur, though it has existed only for a few months, are highly necessary. I shall prescribe, for the present, oat-meal mush and sweet milk, as regular diet, and pustulation over the epigastrum with croton oil. The oil may be rubbed on the skin once or twice a day till the eruption appears sufficiently, and renewed as the pustules disappear. A pint of milk with suitable amount of mush, to be taken three times a day until his return a week hence, will be sufficient.

Although the billiary secretion is probably deficient, mercurial preparations could not prove beneficial, as the liver is already probably irritable and excited, and would not secrete more freely under the additional stimulus of mercury.

Pancreatic and gastric fluids are probably deficient with him, and will be considered at his next visit. They may be artificially afforded in the form of pepsine and pancreatine, and by their regular administration at each meal, relief may be more certainly secured.

